# Trends in E-Cigarette and Tobacco Cigarette Purchasing Behaviors by Youth in the United States, Canada, and England, 2017–2022

**DOI:** 10.3389/ijph.2023.1606234

**Published:** 2023-11-14

**Authors:** Avery Roberson, K. Michael Cummings, Jessica L. Reid, Robin Burkhalter, Shannon Gravely, Katherine East, James F. Thrasher, David Hammond

**Affiliations:** ^1^ Department of Psychiatry & Behavioral Sciences, Medical University of South Carolina, Charleston, SC, United States; ^2^ School of Public Health Sciences, University of Waterloo, Waterloo, ON, Canada; ^3^ Department of Psychology, University of Waterloo, Waterloo, ON, Canada; ^4^ National Addiction Centre, Institute of Psychiatry and Neuroscience, King’s College London, London, England; ^5^ Department of Health Promotion, Education and Behavior, Arnold School of Public Health, University of South Carolina, Columbia, SC, United States

**Keywords:** youth, survey, tobacco, e-cigarettes, minimum legal age

## Abstract

**Objectives:** This paper describes trends in youth e-cigarette (EC) and tobacco cigarette (TC) purchasing behaviors in Canada, England, and the United States (US) in relationship to changing minimum legal age (MLA) laws.

**Methods:** Data are from eight cross-sectional online surveys among national samples of 16- to 19-year-olds in Canada, England, and the US conducted from 2017 to 2022 (N = 104,467). Average wave percentage change in EC and TC purchasing prevalence and purchase locations were estimated using Joinpoint regressions.

**Results:** EC purchasing increased between 2017 and 2022, although the pattern of change differed by country. EC purchasing plateaued in 2019 for the US and in 2020 for Canada, while increasing through 2022 for England. TC purchasing declined sharply in the US, with purchasing from traditional retail locations declining, while purchasing from social sources increased. Vape shops were the most common location for EC purchasing, although declining in England and the US.

**Conclusion:** Trends in EC and TC purchasing trends in the US are consistent with the expected impact of the federal MLA law increasing the legal age to 21 years in December 2019.

## Introduction

Recent studies have revealed shifting patterns in the use of tobacco and e-cigarettes by youth [1–3]. Findings from the ITC Youth Tobacco and Vaping survey show that in the United States (US), past-30-day tobacco cigarette (TC) prevalence had fallen from 11% in 2017 to 3% in 2022, while past-30-day e-cigarette (EC) use increased from 11% to 15% during the same period [3]. Similar trends were seen in Canada, with TC prevalence declining from 11% to 8% and EC use increasing from 8% to 16% between 2017 and 2022 [3]. In England, past-30-day TC prevalence increased from 16% to 21% between 2017 and 2022, while EC prevalence increased 3-fold from 9% to 24% [3].

Minimum legal age (MLA) is a common policy intervention aimed at reducing youths’ legal access to substances such as alcohol, tobacco and cannabis [4–8]. MLA policies make it illegal to sell products to minors and indirectly reduce access by increasing the time and effort required by youth to find alternate sources [4–8]. There is evidence demonstrating that higher MLA for alcohol sales is associated with lower alcohol consumption among youth, although the evidence for tobacco and cannabis is less certain [4, 6–8]. Several recent studies provide suggestive evidence that youth in jurisdictions that adopted MLA laws of ≥21 years for tobacco products had less tobacco use and lower perceived access to tobacco products compared to jurisdictions with MLA of <21 years [9–11]. While the US, Canada and England have restricted tobacco sales to those ≥18 years of age for a number of years, some changes to MLA laws for purchasing tobacco have recently been implemented [4, 6, 12]. The US federal MLA for tobacco products was raised from ≥18 to ≥21 years, effective December 2019, although between 2016 and 2019, 12 states had already implemented this change [6, 12, 13]. In Canada, provinces and territories have a MLA of ≥18 years (4 provinces) or ≥19 years (6 provinces and 3 territories, two of which increased their MLA from ≥18 years of age in 2020), although one province (Prince Edward Island) increased their MLA from ≥19 to ≥21 years in 2020 [6]. In England, the MLA for tobacco products was increased in 2007 from ≥16 to ≥18 years, where it has since remained, although recently England has planned to introduce legislation to prohibit children born on or after 1 January 2009 from legally buying tobacco cigarettes, recent proposals suggest raising the MLA for purchase of cigarettes to ≥21 years [14].

A 2022 survey of young people aged 15–20 years who used ECs in the past-30-days in the US found that 59% reported obtaining their ECs from social sources, while 41% reported purchasing from a retail outlet, with vape shops and gas/convenience stores mentioned most often [15]. We found similar results using data from the 2017 International Tobacco Control (ITC) Youth Tobacco and Vaping Survey in Canada, England, and the US, where 35% of youth between the ages of 16–19 years who had used an EC in the past 12 months had purchased an EC themselves, and vape shops were the most commonly reported purchase location [16]. This study also found that purchasing an EC was more common among older youth (i.e., 18–19 years old vs. 16–17 years old) and among those who reported using an EC and/or smoking TCs more frequently (i.e., 20 of the past-30-day). Retail sources for ECs among youth could reflect different levels of access for “underage sales,” as well as preferences for specific types of ECs, whose availability differs across retail settings. For example, popular pod/cartridge-based ECs were primarily available in gas stations and convenience stores, whereas “tank” devices and refillable e-liquids are most commonly sold in vape shops [6, 16–18].

The current study extends our previous study by evaluating trends in purchasing ECs and TCs among youth aged 16–19 years in Canada, England, and the US between 2017 and 2022 [16]. Comparison across the three countries with varying MLA laws provides an initial assessment of the possible impact of raising the MLA on EC and TC purchasing behaviors by adolescents.

## Methods

### Sample

Data were collected as part of the ITC Youth Tobacco and Vaping Surveys conducted in Canada, England, and the US [3]. In this paper, we report on eight annual and bi-annual repeated cross-sectional online surveys conducted between 2017 and 2022 with national samples of ∼4,500 youths aged 16–19 years in each survey wave in each country (*n* = 104,467). The timing of survey waves were as follows: Wave 1: July/August 2017; Wave 2: August/September 2018; Wave 3: August/September 2019; Wave 3.5: February/March 2020; Wave 4: August 2020; Wave 4.5: February/March 2021; Wave 5: August/September 2021; and Wave 6: August/September 2022. In each country, respondents were recruited by Nielsen Consumer Insights Global Panel via e-mail invitation. Youth aged 16 to 19 were recruited directly or through their parents. Panelists who were parents of children aged 16 to 19 in their household were asked for permission for their child to take the survey. All respondents provided informed consent. The surveys were available in English in all countries, as well as French in Canada. On completion of the survey, respondents received remuneration in accordance with their panel’s usual incentive structure, which could include points-based or monetary rewards and/or chances to win monthly prizes. All surveys were reviewed and received ethics clearance through a University of Waterloo Research Ethics Committee. A full description of the study methods, response rates, and survey questions can be found online [19].

### Measures

Questions about EC purchasing were asked in all survey waves, but TC purchasing questions were not assessed in 2017 or 2018 surveys. Separate, parallel items were asked for each of ECs and TCs.

#### Use of ECs and TCs

All respondents were asked about ever use of ECs and/or TCs, and those who said yes were asked about recency of use, with options for past 12 months and past 30 days. Those who had used in the last 30 days were asked on how many of the past days they had used; use on at least 20 of 30 days was considered “regular use.”

#### Purchasing Behaviors and Locations of Purchase

Respondents who had used an EC or TC in the past 12 months were asked if they purchased an EC or TC in the past 12 months. Among those who had purchased in the past 12 months, purchase locations were assessed using a multiple selection list with seven options, as well as “don’t know” and “refused.” Purchase locations for ECs and TCs were re-coded into four separate outcomes (since respondents could report multiple locations): 1) specialty tobacco/vape shops (“vape shop” or “tobacconist”), 2) traditional retail stores (“regular store/shop such as a convenience/gas station, supermarket, etc.” or “chemist/pharmacy”), 3) the internet, and 4) social sources (“friend or family member” or “someone else”).

### Analyses

Analyses were restricted to those who reported past-12-month EC and/or TC use, or both in the case of dual users. Post-stratification sample weights were calculated for each country, based on age, sex, geographic region, and race/ethnicity (US only). In addition, subsequent survey waves were calibrated back to Wave 1 proportions for student status (student vs. not) and school grades (<70%, don’t know, and refused; 70%–79%; 80%–89%; 90%–100%) and used the National Youth Tobacco Survey (NYTS) in the US, and the Canadian Student Tobacco, Alcohol and Drugs Survey (CSTADS) in Canada to calibrate to the trend over time for smoking in the last 30 days. Participants were excluded from the dataset if they failed a data integrity check, in which they were asked to select the current month from a list and were excluded from the analytic sample if not classified for sex, smoking status, or vaping status variables.

Descriptive weighted analyses assessed EC and TC purchasing and purchase locations among past-12-month product users. We used Joinpoint regressions to test for trends in outcome variables assessed between 2017 and 2022 [20]. We report the average wave percent change (AWPC) and 95% confidence intervals for each outcome to determine if the average trend is significantly different than zero at the alpha ≤0.05 level. Joinpoint regression also allows for breaking the data into different time segments (i.e., joinpoints) to identify survey wave differences that may differ significantly. To examine participant characteristics associated with product purchasing and purchase locations, we conducted logistic regression models using the respondents’ sex, age (16–17 years vs. 18–19 years), and frequency of product use (i.e., “regular use” defined as use ≥20 of the past 30 days vs. “less than regular use” defined as <20 of the past 30 days) as predictor variables for each product (EC vs. TC), stratified by country. All analyses were conducted in SAS V.9.4 (SAS Institute, Cary, NC).

## Results

Table 1 shows EC purchasing and purchase locations for youth who had used an EC in the prior 12 months by country. EC purchasing increased in all three countries between 2017 and 2022. Across all countries and survey waves, vape shops and traditional retail stores that also sell cigarettes (e.g., convenience stores, gas stations) were the primary purchase locations reported by youth.

**TABLE 1:** Weighted percentage of e-cigarette purchasing/purchase locations among past 12-month users by country and survey wave (International Tobacco Control Youth Tobacco and Vaping Study, Canada, England, and the United States, 2017–2022).

Year and month of survey	2017_Aug_	2018_Aug_	2019_Aug_	2020_Feb_	2020_Aug_	2021_Feb_	2021_Aug_	2022_Aug_
**Canada**	4,038	3,845	4,135	4,217	4,269	4,611	4,604	4,395
Total sample size per wave								
Have you bought an e-cigarette in the past 12 months?^a^	31.9%	37.8%	46.1%	46.8%	46.0%	57.9%	54.5%	55.8%
Purchase locations reported among those who bought an e-cigarette in past 12 months^b^								
• % Vape shop	68.3%	63.9%	62.2%	61.4%	58.2%	61.6%	60.7%	64.4%
• % Traditional retail store	24.9%	17.5%	25.6%	31.1%	35.7%	35.9%	36.6%	33.1%
• % Internet	19.5%	25.1%	13.2%	10.5%	14.4%	9.3%	13.0%	11.6%
• % Friend/family/other	NA	28.7%	32.9%	33.4%	33.0%	33.1%	30.1%	34.0%
**England**	3,995	3,874	3,493	4,275	4,290	4,298	4,316	4,283
Total sample size per wave								
Have you bought an e-cigarette in the past 12 months?^a^	26.1%	36.9%	40.5%	46.3%	47.1%	47.1%	51.5%	60.7%
Purchase locations reported among those who bought an e-cigarette in past 12 months^b^								
• % Vape shop	57.6%	58.3%	53.2%	55.8%	49.7%	45.4%	48.8%	47.3%
• % Traditional retail store	27.2%	34.0%	35.8%	39.4%	40.2%	36.3%	44.3%	64.7%
• % Internet	27.8%	26.6%	29.1%	23.0%	29.0%	33.1%	27.1%	16.0%
• % Friend/family/other	NA	17.3%	23.0%	19.4%	19.8%	22.7%	22.6%	20.5%
**United States**	4,095	4,034	3,981	5,132	5,991	5,273	4,881	4,142
Total sample size per wave								
Have you bought an e-cigarette in the past 12 months?^a^	39.6%	52.3%	53.3%	56.8%	52.1%	53.5%	54.2%	52.9%
Purchase locations reported among those who bought an e-cigarette in past 12 months^b^								
• % Vape shop	64.8%	61.3%	55.4%	54.4%	50.1%	53.2%	56.0%	51.8%
• % Traditional retail store	34.5%	36.9%	42.2%	46.6%	45.3%	39.0%	39.2%	36.9%
• % Internet	20.5%	26.8%	17.5%	14.8%	16.5%	16.0%	12.0%	9.5%
• % Friend/family/other	NA	24.9%	30.5%	38.9%	38.9%	35.2%	37.1%	45.0%

NA, not assessed.

^a^ Percentage among past 12-month e-cigarette users.

^b^ Percentage of past 12-month users who also reported buying an e-cigarette for themselves in the past year.

Table 2 shows TC purchasing and purchase location for youth who had used TCs in the prior 12 months. Between 2019 and 2022, TC purchasing was relatively stable in Canada and England, but declined among US youth. Across all survey waves, traditional retail stores were the primary location for purchasing TCs in all three countries.

**TABLE 2:** Weighted percentage of tobacco cigarette purchasing/purchase locations among past 12-month users by country and survey wave (International Tobacco Control Youth Tobacco and Vaping Study, Canada, England, and the United States, 2017–2022).

Year and month of survey	2017_Aug_	2018_Aug_	2019_Aug_	2020_Feb_	2020_Aug_	2021_Feb_	2021_Aug_	2022_Aug_
**Canada**	4,038	3,845	4,135	4,217	4,269	4,611	4,604	4,395
Total sample size per wave								
Have you bought cigarettes in the past 12 months?^a^	NA	NA	46.2%	43.5%	45.1%	45.5%	44.0%	38.2%
Purchase locations reported among those who bought cigarettes in past 12 months^b^	NA	NA						
• % Tobacco specialty shop			16.5%	12.5%	14.3%	12.5%	17.2%	16.3%
• % Traditional retail store			77.9%	75.9%	78.4%	77.9%	74.6%	74.9%
• % Internet			5.1%	3.8%	7.2%	3.6%	8.1%	6.9%
• % Friend/family/other			23.8%	29.4%	25.6%	25.3%	32.5%	28.3%
**England**	3,995	3,874	3,493	4,275	4,290	4,298	4,316	4,283
Total sample size per wave								
Have you bought cigarettes in the past 12 months?^a^	NA	NA	47.4%	52.7%	51.9%	54.6%	49.4%	55.7%
Purchase locations reported among those who bought cigarettes in past 12 months^b^	NA	NA						
• % Tobacco specialty shop			18.9%	21.6%	16.6%	12.5%	20.7%	29.8%
• % Traditional retail store			80.1%	83.4%	85.7%	86.6%	84.1%	90.0%
• % Internet			5.9%	3.7%	5.4%	3.2%	7.4%	7.6%
• % Friend/family/other			23.1%	20.2%	18.6%	20.0%	21.3%	18.9%
**United States**	4,095	4,034	3,981	5,132	5,991	5,273	4,881	4,142
Total sample size per wave								
Have you bought cigarettes in the past 12 months?^a^	NA	NA	44.8%	43.5%	41.8%	35.7%	27.9%	23.8%
Purchase locations reported among those who bought cigarettes in past 12 months^b^	NA	NA						
• % Tobacco specialty shop			17.6%	14.0%	16.6%	16.0%	17.3%	13.6%
• % Traditional retail store			82.7%	75.6%	74.6%	70.9%	68.1%	69.1%
• % Internet			8.5%	6.0%	15.7%	8.7%	18.4%	8.7%
• % Friend/family/other			23.9%	30.6%	31.8%	30.4%	33.9%	53.4%

NA, not assessed.

^a^ Percentage among past 12-month cigarette users.

^b^ Percentage of past 12-month cigarette users who also reported buying cigarettes for themselves in the past year.

### Trend Analyses

**FIGURE 1:**
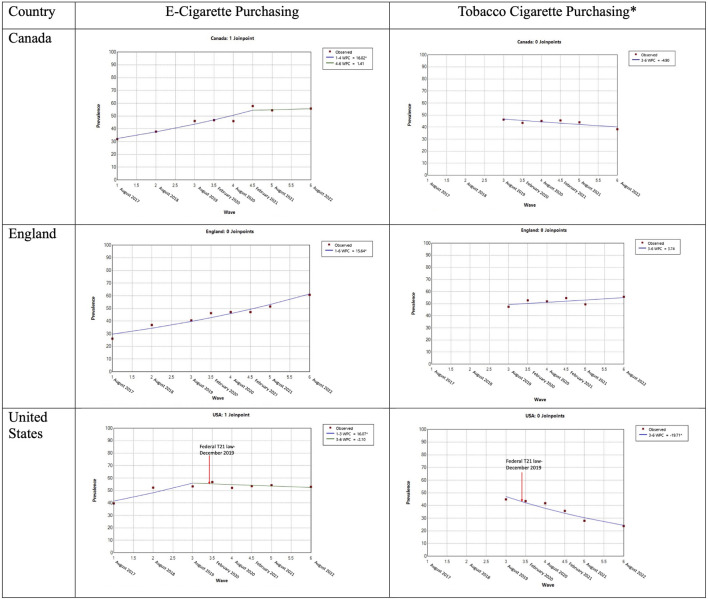
Joinpoint plots showing purchasing trends by purchase location for e-cigarettes and tobacco cigarettes among past 12 months users by country (International Tobacco Control Youth Tobacco and Vaping Study, Canada, England, and the United States, 2017–2022).

Table 3, Figure 1 and Supplementary Figures S2A–D summarize the results of Joinpoint trend analyses for EC and TC purchasing and purchase locations between 2017 and 2022.

**TABLE 3:** Joinpoint regressions testing for trends in e-cigarette and tobacco cigarette purchasing, and purchase locations between 2017 and 2022 by country (International Tobacco Control Youth Tobacco and Vaping Study, Canada, England, and the United States, 2017–2022).

Product	Canada		England		United States	
	E-Cigarettes	Tobacco cigarettes	E-Cigarettes	Tobacco cigarettes	E-Cigarettes	Tobacco cigarettes
Outcome Variables	AWPC+ (95% CI)	AWPC (95% CI)	AWPC (95% CI)	AWPC (95% CI)	AWPC (95% CI)	AWPC (95% CI)
Product Purchasing	11.7%* (6.7% to 16.9%)	−4.9% (−9.7% to 0.1%)	15.6%* (11.3% to 0.2%)	3.7% (−2.5% to 10.3%)	4.8%* (1.3% to 8.5%)	−19.7%* (−26.1% to −12.7%)
Purchase Locations						
Vape shop/tobacco specialty shop	−1.1% (−2.9% to 0.8%)	3.7% (−10.2% to 19.7%)	−4.6%* (−6.8% to −2.3%)	12.5% (−13.8% to 46.7%)	−4.9%* (−7.0% to −2.8%)	−7.5% (−16.7% to 2.7%)
Traditional retail store	11.4%* (0.8% to 23.2%)	−2.2%* (−4.4% to 0.0%)	15.5%* (9.2% to 22.2%)	2.2% (−0.5% to 5.1%)	1.2% (−2.8% to 5.3%)	−6.4%* (−9.4 to −3.3)
Internet	−14.6%* (−25.8% to-1.8%)	−14.6%* (−25.8% to −1.8%)	−1.1 (−9.2% to 7.8%)	12.9% (−18.0% to 55.6%)	−13.3%* (−22.3% to −3.2%)	1.9% (−37.9% to 67.2%)
Friend/family/other	0.5% (−3.8% to 5.0%)	0.4% (−15.0% to 18.6%)	3.1% (−7.0% to 14.3%)	−7.3% (−16.4% to 2.8%)	9.1% (−9.1% to 31.0%)	15.7%* (7.8% to 24.2%)

+AWPC, average wave percentage change.

*Statistically different from zero at the *p* < 0.05 level.

### EC Purchasing

As shown in Table 3 and Figure 1, in each of the three countries, prevalence of EC purchasing among youth who had used ECs in the past 12 months increased between 2017 and 2022. However, the pattern of change differed by country, with increasing EC purchasing plateauing in 2019 in the US (1–3 WPC = 16.7%, *p* < 0.05) and in 2020 in Canada (1–4 WPC = 16.0, *p* < 0.05), but increasing through 2022 in England (AWPC 15.6%, *p* < 0.05).

### EC Purchase Locations

As shown in Table 3 and Supplementary Figures S2A–D, purchasing from vape shops declined significantly in England (AWPC −4.6%, 95% CI, −6.8% to −2.3%) and the US (AWPC −4.9%, 95% CI, −0.7% to −2.8%), but not Canada (AWPC −1.1%, 95% CI, −2.9% to 0.8%). Traditional retail stores purchasing increased for ECs among youth in Canada (AWPC 11.4%, 95% CI, 0.8% to 23.2%) and England (AWPC 15.5%, 95% CI, 9.2% to 22.2%), but not in the US (AWPC 1.2%, 95% CI, −2.8% to 5.3%). The internet was a less common purchase location for ECs (see Table 1), with significant declines between 2017 and 2022 in Canada (AWPC −14.6, 95% CI, −25.8% to −1.8%) and the US (AWPC −13.3%, 95% CI, −22.3% to −3.2%), but not England (AWPC −1.1%, 95% CI, −.9.2% to 7.8%).

### TC Purchasing

As shown in Table 3 and Figure 1, among youth who had used TCs in the prior 12 months, the trends in the prevalence of purchasing TCs between 2019 and 2022 differed by country, with a −19.7% decline in the US (95% CI, −26.1% to −12.7%), but no significant change in England (AWPC 3.7%, 95% CI, −2.5% to 10.3%) or Canada (AWPC −4.9%, 95% CI, −9.7% to 0.1%).

### TC Purchase Locations

As shown in Table 3 and Supplementary Figures S2A–D, TC purchasing locations reported by youth in Canada declined between 2019 and 2022 for traditional retail outlets (AWPC −2.2%, 95% CI, −4.4% to 0.0%) and the internet (AWPC −14.6%, 95% CI, −25.8% to −1.8%). In the US, purchasing locations for TCs declined between 2019 and 2022 for traditional retail stores (AWPC −6.4%, 95% CI, −9.4% to −3.3%), and increased for social sources (AWPC 15.7%, 95% CI, 7.8% to 24.2%). In England, there were no significant changes in TC purchase locations.

### Correlates of EC and TC Purchasing and Purchase Locations

Table 4 summarizes the results of logistic regressions examining the characteristics of respondents associated with EC and TC purchasing by country. Both EC and TC purchasing were associated with being older (18–19 years vs. 16–17 years), male, and vaping and/or smoking regularly. The associations observed were similar in all three countries.

**TABLE 4:** Predictors of e-cigarettes and tobacco cigarette purchasing among past 12-month e-cigarette and tobacco cigarette users by country- all survey waves combined (International Tobacco Control Youth Vaping and Tobacco Study, Canada, England, and the United States, 2017–2022).

Country	Canada		England		United States	
Predictor variables	EC (*n* = 10,554)	TC (*n* = 7,902)	EC (*n* = 9,940)	TC (*n* = 10,297)	EC (*n* = 11,267)	TC (*n* = 7,013)
Age						
16–17 years	ref	ref	ref	ref	ref	ref
18–19 years	1.4 (1.31–1.56)***	1.8 (1.60–2.10)***	1.5 (1.32–1.61)***	1.6 (1.44–1.82)***	1.4 (1.25–1.57)***	1.6 (1.32–1.86)***
Sex						
Male	ref	ref	ref	ref	ref	ref
Female	0.8 (0.70–0.84)***	0.6 (0.59–0.77)***	0.7 (0.66–0.80)***	0.7 (0.65–0.82)***	0.7 (0.62–0.78)***	0.6 (0.51–0.70)***
Regular EC use						
<19 days out of 30 days	ref	ref	ref	ref	ref	ref
≥20 days out of 30 days	30.1 (24.3–37.2)***	1.2 (1.05–1.45)**	34.2 (25.0–46.8)***	2.5 (2.06–3.01)***	30.9 (23.3–41.0)***	1.5 (1.20–1.80)***
Regular TC use						
<19 days out of 30 days	ref	ref	ref	ref	ref	ref
≥20 days out of 30 days	2.3 (1.92–2.75)***	17.0 (13.1–22.0)***	2.6 (2.26–3.00)***	17.4 (13.9–1.6)***	3.2 (2.60–4.00)***	18.1 (13.2–24.7)***

*p < 0.05; **p < 0.01; ***p < 0.001.

Table 5 summarizes the results of logistic regressions examining the characteristics of respondents associated with EC and TC purchase location by country. Older respondents (18–19 years vs. 16–17 years) and those who vaped and/or smoked regularly were more likely to report buying ECs and TCs from traditional retail stores and buying ECs from a vape shop. Younger respondents were more likely to report buying ECs and TCs from social sources and buying TCs from the internet. Those who reported buying ECs from a vape shop were less likely to also report buying ECs in other locations. Respondents who reported buying TCs from a tobacconist were more likely to also report buying TCs from the internet, and vice versa.

**TABLE 5:** Predictors of e-cigarettes and tobacco cigarette purchase locations among past 12-month e-cigarette and tobacco cigarette users by country- all survey waves combined (International Tobacco Control Youth Vaping and Tobacco Study, Canada, England, and the United States, 2017–2022).

Country	Canada		England		United States	
Predictors by location	EC (*n* = 10,554)	TC (*n* = 7,092)	EC (*n* = 9,940)	TC (*n* = 10,297)	EC (*n* = 11,267)	TC (*n* = 7,013)
Vape shop/tobacco shop						
Age						
16–17 years	ref	ref	ref	ref	ref	ref
18–19 years	2.0 (1.77–2.37)***	1.3 (0.94–1.80)	1.3 (1.16–1.57)***	0.7 (0.59–0.89)*	1.6 (1.38–1.88)***	1.0 (0.70–1.39)
Sex						
Male	ref	ref	ref	ref	ref	ref
Female	1.2 (1.06–1.40)**	0.6 (0.48–0.82)***	0.9 (0.79–1.05)	0.7 (0.56–0.80)***	1.1 (0.92–1.24)	0.9 (0.68–1.29)
Regular EC use						
<19 days out of 30 days	ref	ref	ref	ref	ref	ref
≥20 days out of 30 days	2.7 (2.28–3.14)***	1.0 (0.72–1.48)	2.1 (1.74–2.50)***	0.8 (0.63–1.11)	2.2 (1.82–2.54)***	1.3 (0.85–1.85)
Regular TC use						
<19 days out of 30 days	ref	ref	ref	ref	ref	ref
≥20 days out of 30 days	1.7 (1.30–2.16)***	1.5 (1.07–1.97)*	1.5 (1.24–1.77)***	2.0 (1.61–2.38)***	1.6 (1.30–2.07)***	1.5 (1.10–2.05)*
Other purchase locations						
Vape shop/tobacco shop	ref	ref	ref	ref	ref	ref
Traditional retail stores	0.3 (0.24–0.34)***	0.1 (0.08–0.19)***	0.3 (0.22–0.31)***	0.2 (0.14–0.25)***	0.4 (0.36–0.51)***	0.3 (0.19–0.55)***
Internet	0.5 (0.35–0.59)***	5.1 (2.53–10.2)***	0.3 (0.24–0.36)***	4.2 (2.76–6.28)***	0.6 (0.46–0.73)***	4.0 (2.57–6.37)***
Social sources	0.2 (0.18–0.25)***	0.3 (0.17–0.47)***	0.4 (0.28–0.45)***	0.6 (0.41–0.80)**	0.3 (0.25–0.36)***	0.4 (0.22–0.65)***
Traditional retail stores						
Age						
16–17 years	ref	ref	ref	ref	ref	ref
18–19 years	1.4 (1.17–1.57)***	2.2 (1.73–2.90)***	1.3 (1.13–1.52)***	1.7 (1.37–2.20)***	1.2 (1.07–1.45)**	1.8 (1.30–2.52)***
Sex						
Male	ref	ref	ref	ref	ref	ref
Female	1.2 (1.01–1.31)*	1.2 (0.91–1.49)	1.3 (1.09–1.43)**	1.1 (0.87–1.38)	1.0 (0.87–1.16)	1.2 (0.92–1.66)
Regular EC use						
<19 days out of 30 days	ref	ref	ref	ref	ref	ref
≥20 days out of 30 days	1.3 (1.15–1.53)***	1.4 (1.02–2.01)*	1.7 (1.47–2.08)***	1.1 (0.76–1.52)	1.5 (1.30–1.76)***	2.0 (1.37–2.98)***
Regular TC use						
<19 days out of 30 days	ref	ref	ref	ref	ref	ref
≥20 days out of 30 days	1.3 (1.08–1.67)**	2.3 (1.64–3.15)***	1.2 (1.04–1.47)*	2.4 (1.82–3.17)***	1.9 (1.55–2.37)***	2.1 (1.50–2.96)***
Other purchase locations						
Traditional retail stores	ref	ref	ref	ref	ref	ref
Vape shop/tobacco shop	0.3 (0.24–0.34)***	0.1 (0.07–0.17)***	0.3 (0.22–0.31)***	0.2 (0.14–0.24)***	0.4 (0.36–0.51) ***	0.3 (0.18–0.50)***
Internet	0.8 (0.65–1.07)	1.9 (0.79–4.35)	0.3 (0.26–0.40)***	0.9 (0.47–1.66)	0.6 (0.50–0.77) ***	0.9 (0.45–1.70)
Social sources	0.4 (0.32–0.48)***	0.1 (0.07–0.13)***	0.4 (0.32–0.52)***	0.1 (0.09–0.16)***	0.4 (0.35–0.50) ***	0.1 (0.06–0.12)***
Internet						
Age						
16–17 years	ref	ref	ref	ref	ref	ref
18–19 years	0.9 (0.80–1.19)	0.6 (0.35–0.87)*	1.1 (0.95–1.32)	0.6 (0.46–0.92)*	0.9 (0.72–1.06)	0.6 (0.42–0.93)*
Sex						
Male	ref	ref	ref	ref	ref	ref
Female	0.7 (0.61–0.87)***	0.6 (0.41–0.93)*	0.9 (0.75–1.01)	0.4 (0.30–0.59)***	0.7 (0.62–0.90)**	0.5 (0.34–0.72)***
Regular EC use						
<19 days out of 30 days	ref	ref	ref	ref	ref	ref
≥20 days out of 30 days	0.9 (0.75–1.12)	0.6 (0.32–1.13)	1.7 (1.37–1.99)***	0.9 (0.55–1.53)	1.0 (0.80–1.21)	0.6 (0.41–0.99)*
Regular TC use						
<19 days out of 30 days	ref	ref	ref	ref	ref	ref
≥20 days out of 30 days	1.9 (1.45–2.39)***	0.8 (0.54–1.33)	1.0 (0.84–1.25)	1.4 (0.98–1.93)	1.5 (1.18–1.95)**	1.2 (0.80–1.67)
Other purchase locations						
Internet	ref	ref	ref	ref	ref	ref
Vape shop/tobacco shop	0.5 (0.35–0.61)***	4.9 (2.99–8.01)***	0.3 (0.23–0.36)***	4.2 (2.85–6.09)***	0.6 (0.45–0.73)***	4.3 (2.83–6.54)***
Traditional retail store	0.9 (0.66–1.11)	1.3 (0.77–2.33)	0.3 (0.26–0.40)***	0.8 (0.48–1.18)	0.6 (0.48–0.77)***	0.8 (0.48–1.38)
Social sources	0.8 (0.57–1.02)	2.6 (1.54–4.40)***	0.6 (0.44–0.76)***	1.6 (1.08–2.38)*	0.7 (0.55–0.93)*	1.6 (0.99–2.69)
Social Sources						
Age						
16–17 years	ref	ref	ref	ref	ref	ref
18–19 years	0.5 (0.45–0.60)***	0.4 (0.34–0.55)***	0.6 (0.50–0.71)***	0.4 (0.36–0.53)***	0.5 (0.44–0.61)***	0.4 (0.32–0.57)***
Sex						
Male	ref	ref	ref	ref	ref	ref
Female	1.5 (1.28–1.69)***	1.3 (1.00–1.57)*	1.0 (0.82–1.15)	1.1 (0.90–1.32)	1.2 (1.01–1.38)*	1.0 (0.79–1.34)
Regular EC use						
<19 days out of 30 days	ref	ref	ref	ref	ref	ref
≥20 days out of 30 days	1.2 (0.99–1.35)	1.2 (0.92–1.61)	0.9 (0.75–1.16)	1.4 (1.07–1.79)*	1.2 (0.99–1.38)	1.4 (1.01–1.95)*
Regular TC use						
<19 days out of 30 days	ref	ref	ref	ref	ref	ref
≥20 days out of 30 days	0.8 (0.62–1.01)	1.4 (1.07–1.81)*	0.9 (0.70–1.09)	1.3 (1.04–1.58)*	0.7 (0.59–0.95)*	1.3 (1.01–1.79)*
Other purchase locations						
Social sources	ref	ref	ref	ref	ref	ref
Vape shop/tobacco shop	0.2 (0.18–0.25)***	0.3 (0.17–0.45)***	0.4 (0.27–0.45)***	0.6 (0.44–0.83)**	0.3 (0.25–0.36)***	0.4 (0.21–0.63)***
Traditional retail store	0.4 (0.33–0.49)***	0.1 (0.07–0.14)***	0.4 (0.31–0.53)***	0.1 (0.10–0.16)***	0.4 (0.35–0.51)***	0.1 (0.06–0.12)***
Internet	0.7 (0.55–0.96)*	3.0 (1.42–6.16)**	0.6 (0.43–0.76)***	1.5 (0.95–2.41)	0.7 (0.57–0.93)*	1.5 (0.85–2.84)

*p < 0.05; **p < 0.01; ***p < 0.001.

## Discussion

We analyzed trends in EC and TC purchasing behaviors by youth users in Canada, England, and the US between 2017 and 2022 and found that prevalence of EC purchasing increased between 2017 and 2022 in each of the three countries. However, the pattern of change differed by country, with increasing EC purchasing plateauing in 2019 in the US and 2020 in Canada, while increasing through 2022 in England. The plateauing of EC purchasing prevalence after 2019 and continuing decline in TC purchasing observed among US adolescents is consistent with the expected effect of raising the MLA from ≥18 to ≥21 years, which was implemented nationally in December 2019. In general, the pattern of change in EC and TC purchasing in Canada was similar to the US, but less pronounced. It is possible that the less dramatic changes in youth EC and TC purchasing observed among Canadian adolescents is that the changes in MLA laws that occurred were in the least populous provinces and the territories and underrepresented in the study sample. By contrast, in England where the MLA law for tobacco remained unchanged at ≥18 years between 2017 and 2022, prevalence of EC and TC purchasing did not change significantly and actually increased slightly over the study period.

Consistent with our previous study of EC purchasing, in all three countries, EC and TC purchasing were associated with older age and regular product use [16]. Purchasing of tobacco products may represent a milestone in the trajectory of a person’s product use, moving from occasional-experimental use to more regular use. Thus, monitoring trends in youth tobacco purchasing might signal changes in regular product use, and be a leading indicator of population level shifts in nicotine dependence. The increasing prevalence of youth EC purchasing is consistent with data from this same study showing an upward trend in youth EC users reporting symptoms of nicotine dependence [3].

Vape shops were the most commonly reported purchase locations for ECs by youth in all three countries but declined significantly as a purchase source in England and the US. Traditional retail outlets increased as a purchase source in Canada and England, but not in the US. The uptick in EC purchasing in traditional retail outlets coincides with the introduction of novel disposable ECs, such as Elf Bar, which are popular among youth and young adults who vape in England and widely available in traditional brick-and-mortar retail outlets [3, 21]. England may be associated with the introduction of Elf Bar, a disposable EC that came into the marketplace in 2022 and was sold primarily in traditional retail outlets [3, 21]. While much has been written about the role of social media in promoting EC use by youth, in this study we found that EC purchasing from the internet declined significantly in Canada and the US, even though overall prevalence of EC purchasing increased [22–24].

TC purchase locations reported by youth did not change much over time in Canada or England. However, in the US, purchasing from traditional retail locations declined, while purchasing from social sources increased, consistent with the possible impact expected from increasing MLA from ≥18 to ≥21 years that had taken place in many US states and localities and was implemented federally in December 2019 [6, 25]. However, varying implementation dates and unknown levels of compliance with MLA laws adopted by states and localities make it difficult to determine to what extent, if any, changes in MLA laws altered tobacco product use and purchasing patterns by adolescents [6–11, 25–27]. Reid et al. [6] recently reported that higher MLA was associated with lower perceived access to tobacco products by youth. However, the impact on actual tobacco product purchasing is less apparent, since we found EC purchasing increasing in all countries over time, although leveling off in later survey waves in the US and Canada.

This study has several limitations. Samples were recruited through consumer panels and were not probability-based, although we had large samples in each country and used weighting to increase comparability to national population estimates. The study is also based on self-reported purchasing behaviors over the previous 12 month period, which is subject to recall error, especially among less frequent product purchasers. That said, our findings on EC purchase locations are consistent with other published studies [15, 16, 28]. Also, this study did not explicitly test how changes in MLA law in different jurisdictions within countries effected purchasing but aimed to test the broader trends observed over time and between countries in purchasing TC and EC among youth. While our findings are generally consistent with the changing MLA laws for purchasing tobacco products that happened in the US, we also recognize that a variety of factors likely influenced the EC and TC purchasing trends observed in this study. These factors may include the COVID-19 pandemic, media reports about the risks and benefits of using different types of tobacco products (e.g., EVALI), the prevalence and type of tobacco products used by friends and family members, price and affordability of products, proximity to retail outlets, enforcement of MLA laws, and industry marketing [29–31].

### Conclusion

Among youth who used ECs, prevalence of EC purchasing increased between 2017 and 2022 in all three countries, although the pattern of change differed by country: increasing EC purchasing plateaued in 2019 in the US and 2020 in Canada, while increasing through 2022 in England. Among youth who smoked cigarettes, TC purchasing declined sharply in the US, with purchasing from traditional retail locations declining while purchasing from social sources increased. The observed trends in EC and TC purchasing in the US are consistent with the December 2019 implementation of MLA law change that raised the legal of sale for tobacco from ≥18 to ≥21 years. The substantial prevalence of youth EC purchasing in all three countries warrants further attention.
