# Supplementary material for: Trends in E-Cigarette and Tobacco Cigarette Purchasing Behaviors by Youth in the United States, Canada, and England, 2017–2022
Source: Int J Public Health. 2023 Nov 14;68:1606234. doi: 10.3389/ijph.2023.1606234 (PMC10682085; doi:10.3389/ijph.2023.1606234)
Supplement: Supplementary file 1 [file DataSheet1.pdf]

Figure 2A-D. Joinpoint plots showing purchasing trends by purchase location for e-cigarettes and tobacco cigarettes among past 12-month users by country (ITC Youth Tobacco and Vaping Study, Canada, England, and the US, 2017-2022).

Panel A: Vape shops for e-cigarettes and tobacco specialty stores for tobacco cigarettes.

<table>
<tr><th>Country</th><th>E-Cigarette – vape shops</th><th>Tobacco Cigarette* — tobacco specialty stores</th></tr>
<tr><td>Canada</td><td>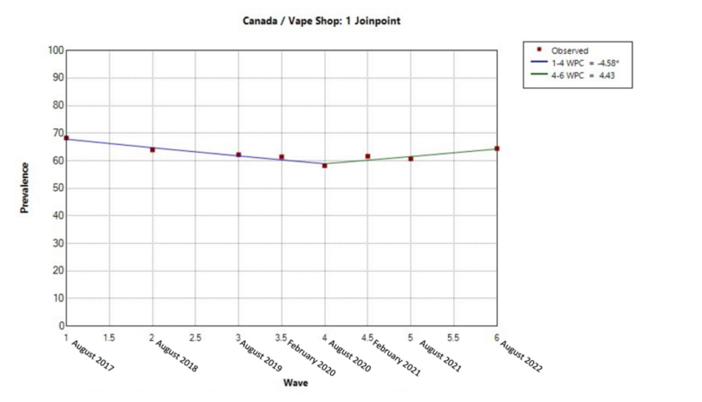
</td><td>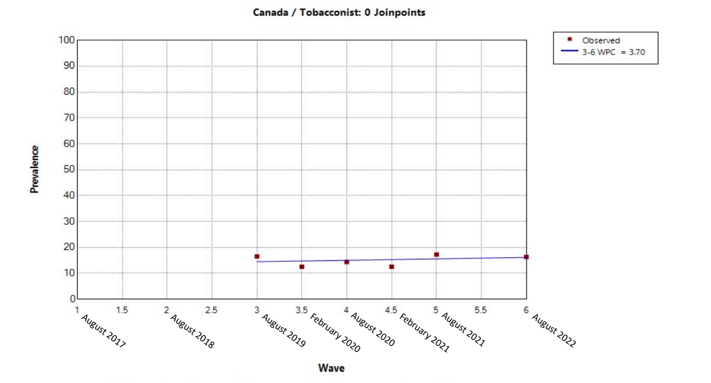
</td></tr>
<tr><td>England</td><td>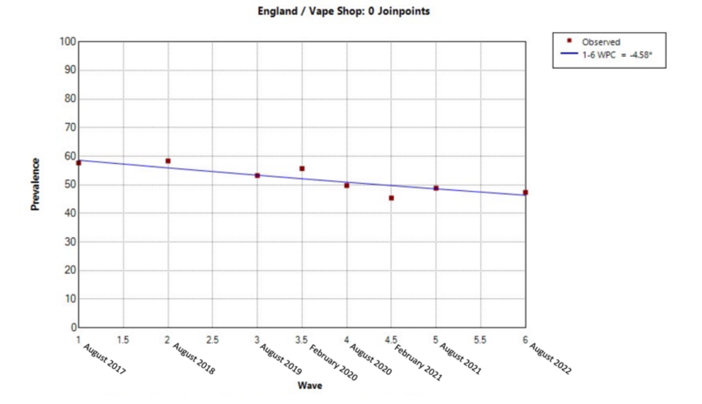
</td><td>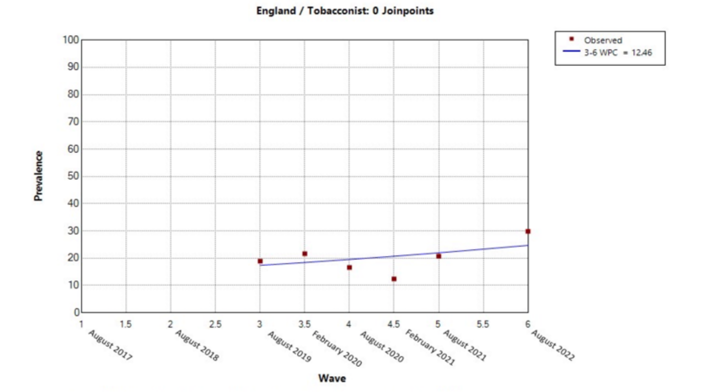
</td></tr>
<tr><td>United States</td><td>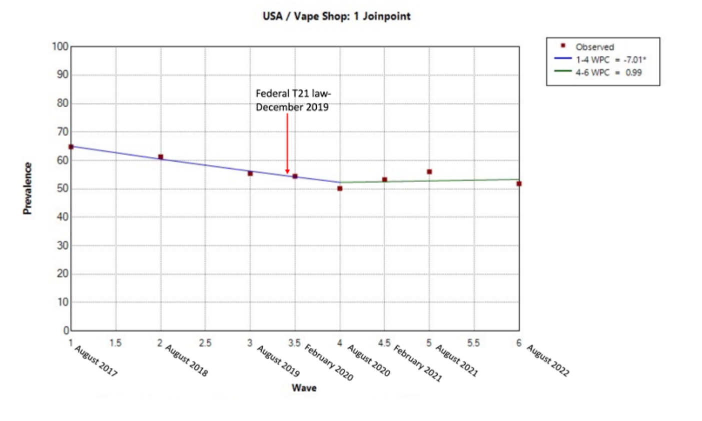
</td><td>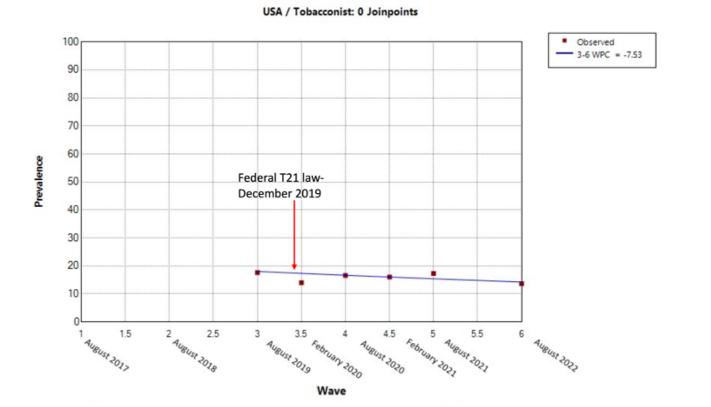
</td></tr>
</table>

Panel B: Traditional retail stores.

Country	E-Cigarettes – traditional retail stores	Tobacco Cigarettes* —traditional retail stores
Canada	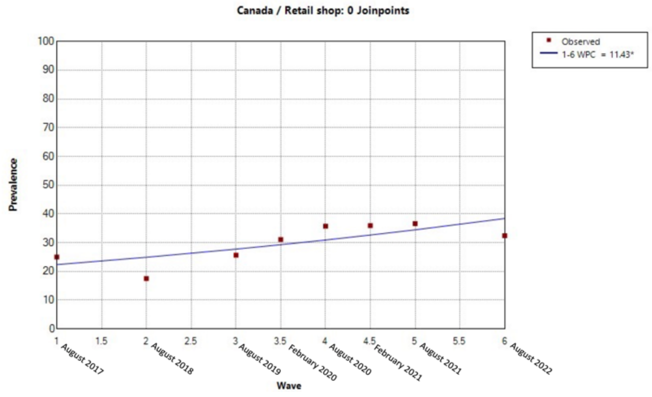	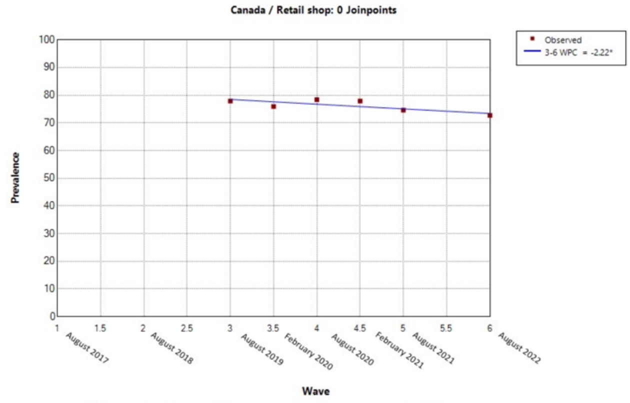
England	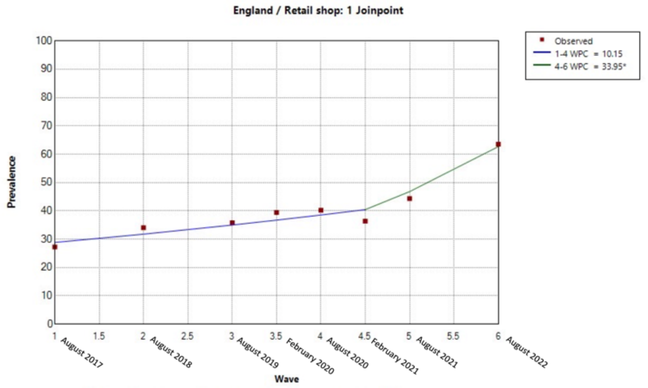	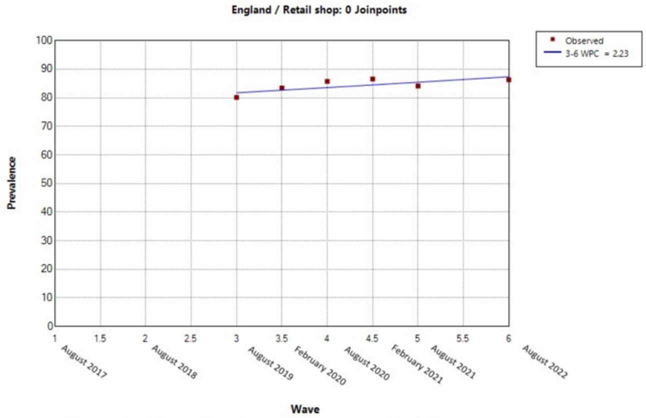
United States	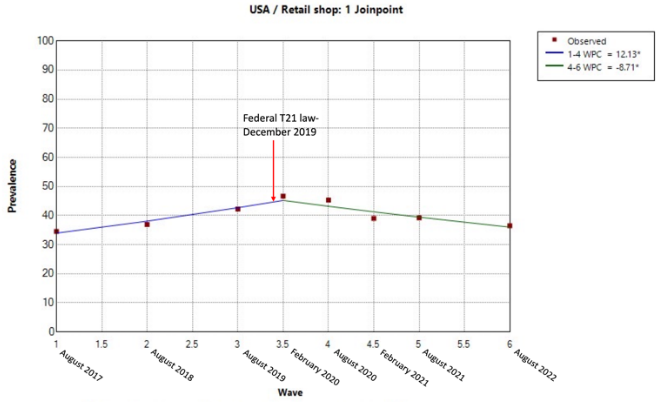	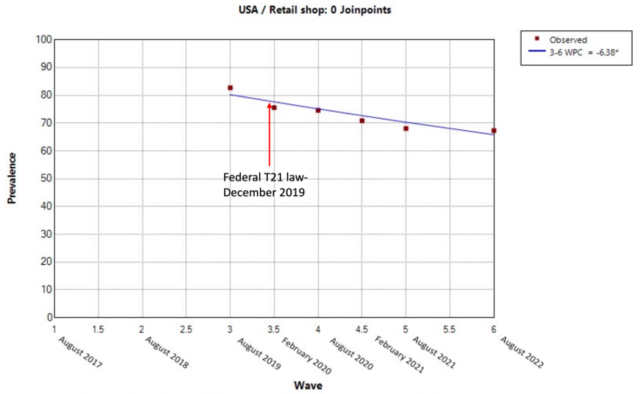

Panel C: Internet.

<table>
<tr><th>Country</th><th>E-Cigarettes – internet</th><th>Tobacco Cigarettes* — internet</th></tr>
<tr><td>Canada</td><td>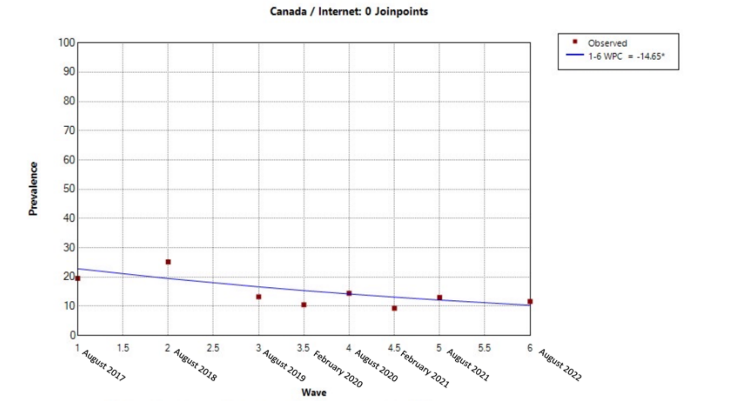
</td><td>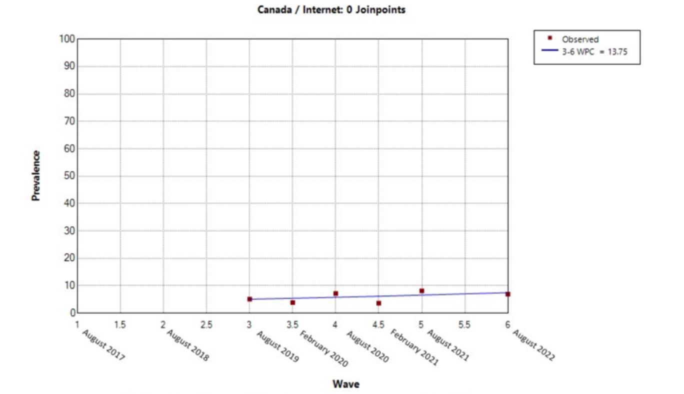
</td></tr>
<tr><td>England</td><td>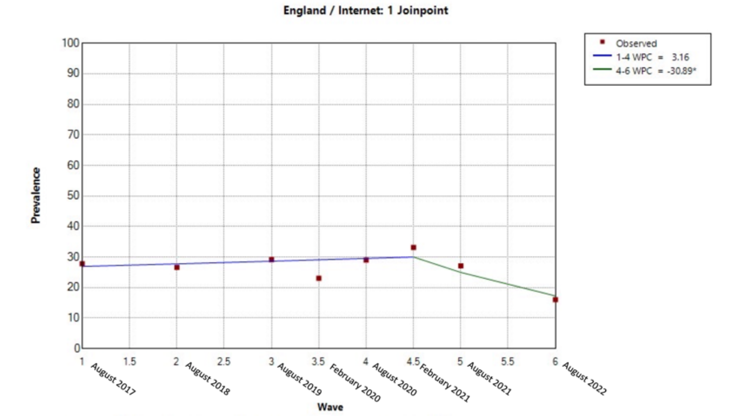
</td><td>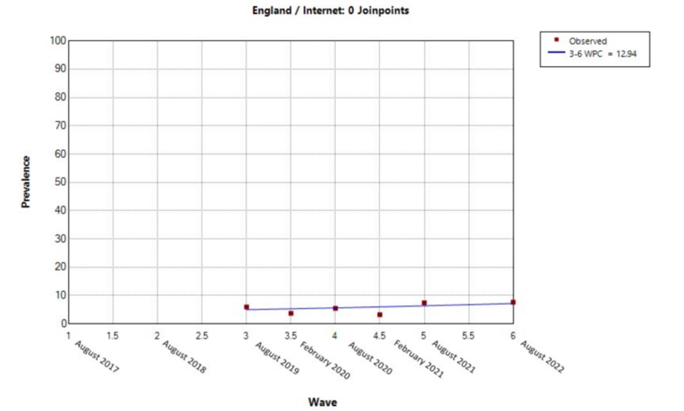
</td></tr>
<tr><td>United States</td><td>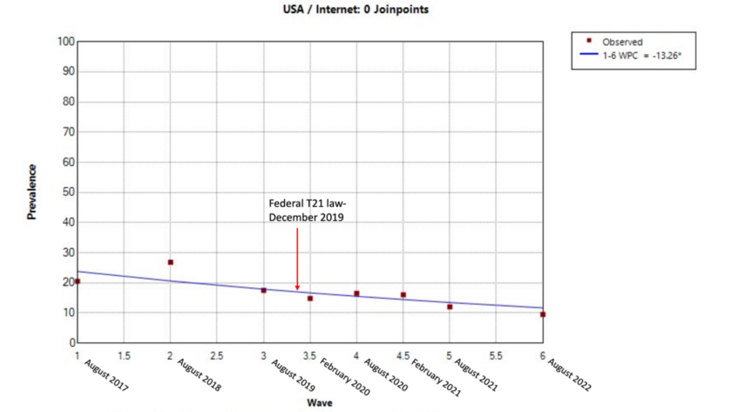
</td><td>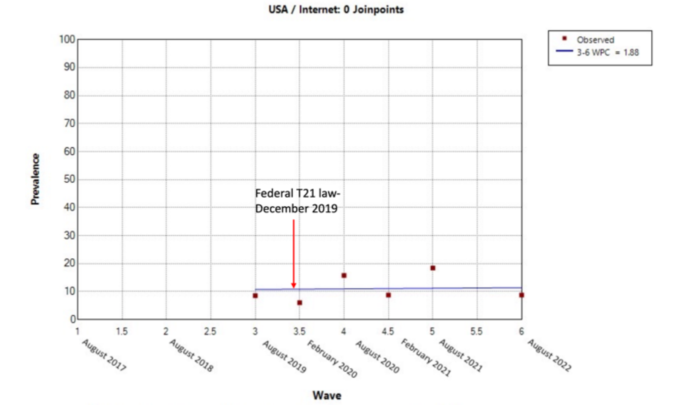
</td></tr>
</table>

Panel D: Social sources.

<table>
<tr><th>Country</th><th>E-Cigarettes – social sources**</th><th>Tobacco Cigarettes* — social sources</th></tr>
<tr><td>Canada</td><td>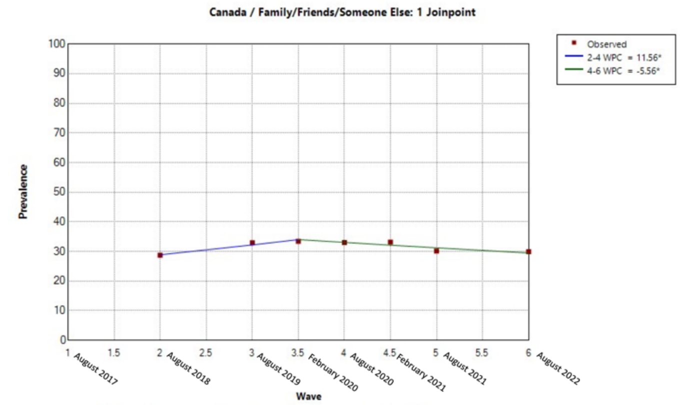
</td><td>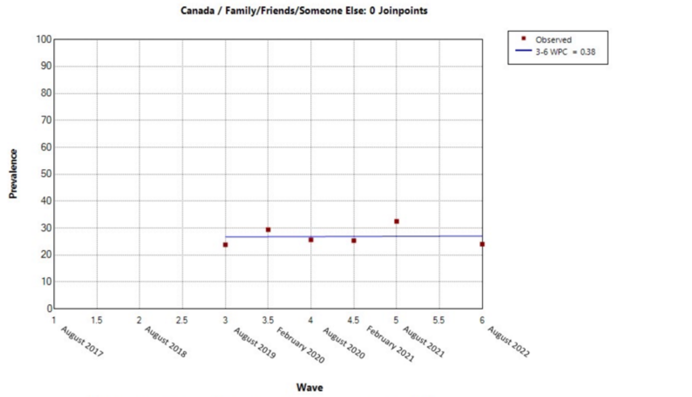
</td></tr>
<tr><td>England</td><td>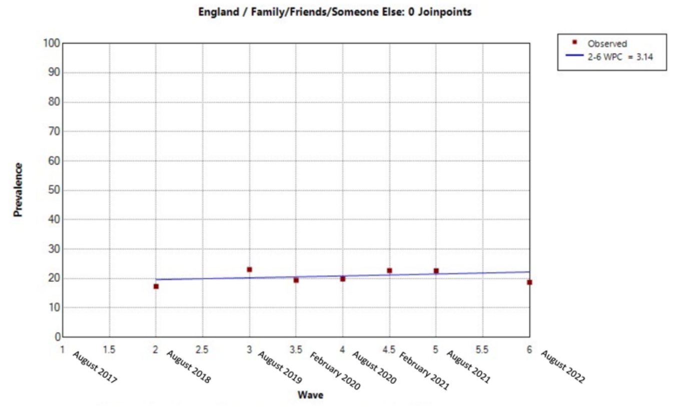
</td><td>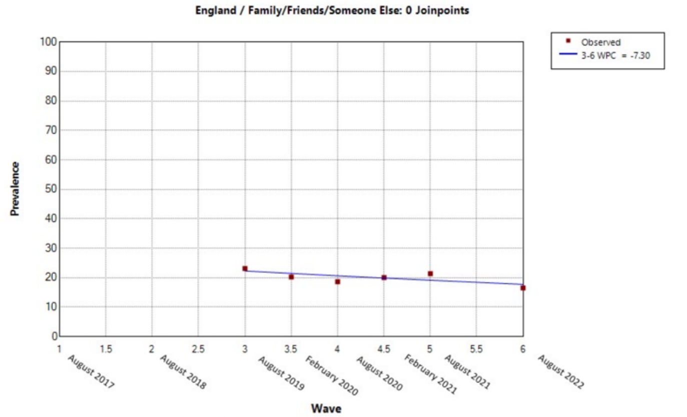
</td></tr>
<tr><td>United States</td><td>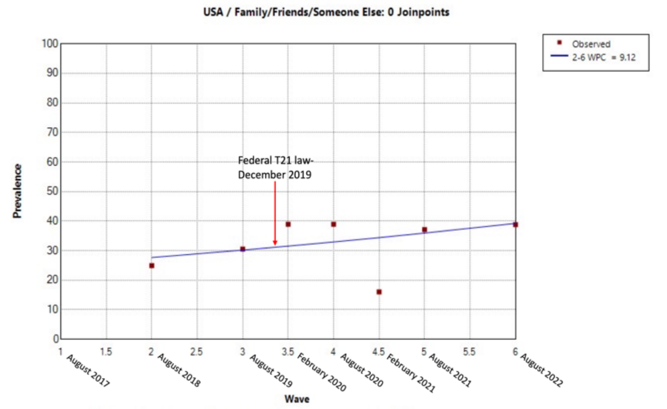
</td><td>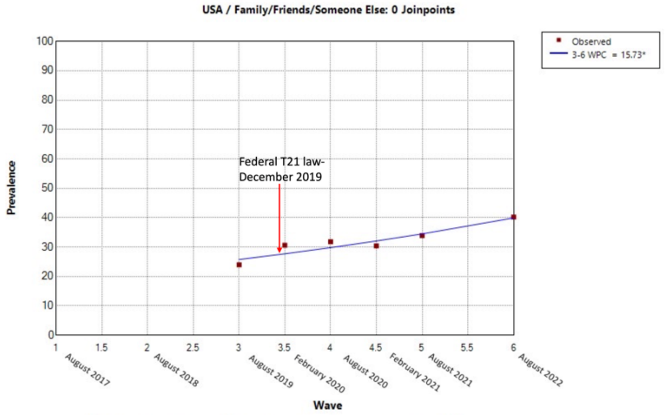
</td></tr>
</table>

*Survey questions regarding tobacco cigarette purchasing were not asked until Wave 3 in August 2019.

**Survey questions regarding e-cigarette purchasing from social sources was not asked until Wave 2 in August 2018.
